# Effects of a 12-Week Exercise Intervention on Primitive Reflex Retention and Social Development in Children with ASD and ADHD

**DOI:** 10.3390/children12080987

**Published:** 2025-07-28

**Authors:** Norikazu Hirose, Yuki Tashiro, Tomoya Takasaki

**Affiliations:** 1 Faculty of Sport Sciences, Waseda University, Tokyo 202-0021, Japan; 2 Gotoschool Inc., Tokyo 150-0043, Japan

**Keywords:** primitive reflex retention, exercise intervention, social behavior, developmental coordination, executive function

## Abstract

**Highlights:**

**What are the main findings?**

• A 12-week exercise program reduced specific primitive reflex retention (e.g., ATNR) and improved fine motor coordination, particularly in children with ASD and ADHD.
• The intervention led to significant behavioral improvements in the ADHD group, as evidenced by reductions in Conners 3 Total and Global Index scores.

**What is the implication of the main finding?**

• Movement-based interventions focusing on rhythm, balance, and coordination may facilitate motor and behavioral development in neurodevelopmental conditions, though effects may vary by diagnosis.
• Such interventions have potential applicability in clinical and educational settings to support self-regulation and developmental outcomes in ASD and ADHD.

**Abstract:**

**Objective:** Retained primitive reflexes are associated with delayed motor and behavioral development in children with autism spectrum disorder (ASD) and attention-deficit/hyperactivity disorder (ADHD). This study examined the effects of a 12-week structured exercise intervention on reflex integration, motor coordination, and socio-behavioral outcomes in these populations. **Method:** Fifteen children with ASD (13 boys, 2 girls) and twelve with ADHD (8 boys, 4 girls), aged 6–12 years, participated in rhythmic, balance, and coordination-based exercises. Primitive reflexes, including the asymmetrical tonic neck reflex (ATNR), were assessed using standardized protocols, and fine motor coordination was evaluated using the Finger and Thumb Opposition Test (FOT). Behavioral outcomes were measured using the Social Responsiveness Scale-2 (SRS-2) for the ASD group and the Conners 3 for the ADHD group. **Results:** The ASD group showed significant reductions in left-standing ATNR retention scores (*p* = 0.012) and improvements in right-hand FOT scores (*p* = 0.023). In the ADHD group, significant improvements were also observed in right-hand FOT scores (*p* = 0.007). Furthermore, Conners 3 Total and Global Index scores significantly decreased in the ADHD group (*p* = 0.016 and 0.020, respectively). Reflex retention patterns appeared broader and more bilateral in ASD than in ADHD, suggesting distinct motor developmental profiles. **Conclusions:** Short-term rhythmic, balance, and whole-body coordination exercise interventions may support behavioral and motor development in children with ASD and ADHD. Tailored programs emphasizing reflex integration hold promise for clinical and educational applications.

## 1. Introduction

### 1.1. Background of ASD and ADHD

In recent years, the prevalence of autism spectrum disorder (ASD) and attention-deficit/hyperactivity disorder (ADHD)—two major neurodevelopmental conditions—has continued to rise. According to the Centers for Disease Control and Prevention, ASD now affects approximately 1 in 31 (3.2%) US children [1]. A recent umbrella review of over 3 million participants estimates the global prevalence of ADHD in children and adolescents at 8% [2], although this varies depending on diagnostic criteria [3].

Both conditions commonly lead to persistent challenges in social communication and motor coordination. Autistic children frequently experience marked social-interaction difficulties that are associated with peer rejection, reduced academic attainment, and lower adult employment rates [4]. Motor impairments are also prevalent, with meta-analyses showing gross- and fine-motor skills averaging over 1 standard deviation below age norms [5,6]. Longitudinal work further suggests that early motor delays forecast later social communication difficulties in ASD [7,8].

Children with ADHD show a parallel profile—academic underachievement, peer conflict, and emotion dysregulation [9,10]—and a high co-occurrence of developmental coordination disorder [11]. A meta-analysis of 55 fMRI studies shows consistent underactivation in the prefrontal cortex and striatum during executive-control tasks in ADHD, with cerebellar involvement trending but non-significant [12]. Long-term follow-up confirms that this combined social–motor burden can persist into adulthood and constrain occupational functioning [13].

### 1.2. The Role of Motor Interventions and Primitive Reflexes

Given the motor challenges, movement-based interventions have shown promise for improving not only coordination but also social and emotional functioning in children with ASD and ADHD. Bhat summarized evidence from multiple studies indicating that motor interventions can enhance social engagement, self-regulation, and adaptive behavior in ASD [7]. Similarly, Bob et al. reported that rhythmic movement activities incorporating musical cues facilitated emotional regulation and prosocial behaviors in autistic children [14].

One hypothesized mechanism underlying these benefits is the integration of retained primitive reflexes (RPRs)—involuntary movement patterns typically inhibited during infancy. Persistence of RPRs beyond early childhood is increasingly recognized as a marker of delayed neurodevelopment [15,16]. Additionally, a cross-sectional study of preschool children demonstrated that those with retained ATNR or STNR scored significantly lower on attention concentration tests, reinforcing the association between RPRs and core ADHD symptoms [17]. RPRs have also been associated with poorer early academic performance [18].

To target these reflexes, intervention programs such as the Primary Movement Program (PMP) [19] and protocols from the Institute for Neuro-Physiological Psychology (INPP) [20] have been developed, focusing on the integration of reflexes such as the asymmetrical tonic neck reflex (ATNR). While quasi-experimental studies have reported reductions in ATNR persistence and associated gains in academic and motor outcomes, systematic reviews highlight a lack of high-quality randomized controlled trials [21].

Building on this framework, we hypothesize that rhythmic, balance, and whole-body coordination exercises can stimulate vestibular and proprioceptive pathways, which are critical for postural control, sensorimotor integration, and cortical maturation [22]. By engaging these systems, such interventions may facilitate reflex integration and, in turn, enhance social engagement, attention, and behavioral regulation.

Nevertheless, few studies have systematically examined whether changes in RPRs expression mediate developmental outcomes, particularly in diagnosed neurodevelopmental groups such as ASD and ADHD.

### 1.3. Role of Fine Motor Assessment and Reflex Integration

In addition to reflex integration, fine motor coordination is a critical area of development in children with ASD and ADHD. Difficulties in fine motor planning and execution have been shown to correlate with poorer academic performance and limitations in daily living skills [23]. Furthermore, RPRs such as the ATNR may interfere with hand-eye coordination and fine motor control during tasks like writing [20,24], suggesting that reflex integration might support broader improvements in motor coordination.

The Finger and Thumb Opposition Test (FOT), while having limited standardization, has been frequently utilized in motor development research as a simple and pragmatic measure of fine motor coordination [24]. Accordingly, the present study incorporated FOT performance alongside primitive reflex assessments to evaluate broader changes in sensorimotor integration.

### 1.4. Current Gaps

Despite the growing interest in RPRs integration, no study has systematically compared the profiles of RPRs between children with ASD and ADHD, nor examined whether a shared motor intervention elicits diagnosis-specific changes in social or executive functioning. While motor proficiency is known to correlate with social–emotional development, the potential mediating role of RPRs integration remains largely unexplored.

Neurodevelopmentally, ASD is characterized by frontal–posterior network underconnectivity [25], whereas ADHD involves immature prefrontal control and striatal dysfunction [12]. This divergence suggests that the mechanisms through which RPRs reduction might influence behavior could differ between these conditions. Furthermore, few studies include objective fine motor assessments, such as the FOT, alongside RPRs measures, limiting insights into functional relevance.

### 1.5. Purpose and Hypotheses

Addressing these gaps, this pilot study examined whether a 12-week rhythmic, balance, and coordination program would (i) reduce overall primitive reflex retention—particularly ATNR—and (ii) improve social behavior (ASD) or executive/behavioral regulation (ADHD). While not powered to formally test mediation, this study explores whether reflex integration may tentatively co-occur with functional improvements in neurodevelopmental populations.

## 2. Methods

### 2.1. Research Design

This study employed a longitudinal pretest–posttest design with four repeated measurement points: baseline (Pre), Week 4 (4W), Week 8 (8W), and Week 12 (12W). Primitive reflexes and fine motor coordination, assessed using the FOT, were measured at all four time points to examine temporal patterns of change in response to a 12-week rhythmic, balance, and coordination intervention among children with ASD and ADHD. In contrast, social and executive functioning were assessed only at baseline (Pre) and at the end of the intervention (12W) to evaluate pre–post changes.

Social and behavioral assessments were conducted only at baseline and 12 weeks, rather than at all measurement points, to minimize respondent burden and because behavioral adaptations typically manifest over longer timescales than motor changes. Additionally, the SRS-2 typically references behaviors over the past six months; however, to align with the 12-week intervention period and accurately capture short-term changes, we adjusted the reference period to three months. This modification ensured that the assessments specifically reflected the intervention period without temporal overlap between pre- and post-measurements.

### 2.2. Participants

Participants were recruited from a pediatric motor therapy center that provides neurodevelopmental support services through physical activity programs. Children aged 6 to 12 years were included, as this age range reflects a critical period for both motor development and the potential integration of retained primitive reflexes while also aligning with the onset of social challenges and school-based adaptation. Inclusion criteria were as follows: (1) children diagnosed with ASD or ADHD by a pediatrician, (2) children who could attend the facility at least once a week and continue participation for the entire 12-week study period, or, if attendance was not possible, could follow the prescribed program at home, and (3) children and their guardians who provided consent to participate in the study. The exclusion criteria were as follows: (1) children who received a dual diagnosis of both ASD and ADHD by a pediatrician, (2) children with musculoskeletal disorders that would interfere with exercise or assessments, (3) children or their guardians who did not consent to participate in this study, (4) children with intellectual developmental delays that prevent them from understanding the assessment procedures, and (5) children who are continuously taking psychotropic medication.

A total of 21 children with ASD and 15 children with ADHD met the above criteria. However, at the beginning of this study (pre), six children from the ASD group and two children from the ADHD group withdrew from participation. Furthermore, during the study period, one child from the ADHD group withdrew due to family circumstances. As a result, the final number of participants who completed the 12-week study was 15 children in the ASD group (13 boys, two girls) and 12 children in the ADHD group (eight boys, four girls) (Table 1).

### 2.3. Assessment Procedure of Primitive Reflex

The presence and degree of primitive reflex retention were assessed using established procedures derived from Blythe [24], which have been applied in recent validation studies of reflex-based interventions [15].

1. The Moro reflex (Moro);
2. TLR Erect test (for flexion; TLR-Flex, for extension; TLR-Ext);
3. STNR (for flexion; STNR-Flex, for extension; STNR-Ext);
4. Ayres quadruped test for the ATNR (ATNR-Quad-L/R for left and right side);
5. Adapted Hoff–Schilder test for the ATNR (ATNR-Stand-L/R for left and right side).

In addition to the above reflexes, we evaluated the following primitive reflexes.

6. Babinski reflex (Babinski);
7. Landau reflex (Landau);
8. Galant reflex (Galant).

In addition to primitive reflex testing, fine motor coordination was assessed via the FOT, as deficits in fine motor control are often co-occurring with retained reflexes and may provide supplementary indicators of functional development, following Blythe’s procedure [24].

All video recordings were captured using a front-facing smartphone, ensuring that participants filled at least 80 % of the frame. To ensure assessment validity, two healthcare professionals (i.e., certified athletic trainers) with expertise in developmental motor analysis (NH and YT) independently evaluated eight randomly selected recordings. Inter-rater reliability was high across the primitive reflex assessments (Moro, TLR, STNR, quadruped-ATNR, and stand-ATNR), with two-way random, absolute-agreement ICC (2,1) values ranging from 0.81 to 0.95. Discrepancies were resolved through consensus discussions. After agreement was reached, the remaining videos were evaluated by NH. Detailed protocols of assessments are described in Supplementary Materials S1.

### 2.4. Evaluation of Social Behavior and Behavioral Characteristics

To assess social and behavioral functioning, caregivers completed standardized questionnaires appropriate to their child’s diagnosis: the Japanese version of the Social Responsiveness Scale, Second Edition (SRS-2) for children with ASD and the Japanese version of the Conners 3 Parent Rating Scale for children with ADHD.

To capture short-term effects of the 12-week intervention, the SRS-2 reference period was shortened from six months to three months to avoid overlap between pre- and post-intervention timeframes. The Conners 3 followed its standard one-month reference period.

Caregivers were provided with verbal instructions by trained researchers on how to complete the forms, including guidance on relevant timeframes and observation points. All assessments were completed on paper, and researchers addressed any questions as needed. For quality control, validity indices (e.g., response consistency) in the Conners 3 were reviewed to ensure reliable responses.

Both instruments have demonstrated strong psychometric properties, with internal consistency above α = 0.85 and test–retest reliability exceeding r = 0.80 in original validation studies [26,27]. Standardized scoring procedures were applied.

### 2.5. Intervention

All participants engaged in both center-based and home-based implementation of the program throughout the 12-week period. Participants attended center-based sessions with a mean of 1.2 sessions week^−1^ (range 0.5–2.4). When attendance fell below one session week^−1^, caregivers were asked to deliver a brief home program and report compliance to the instructor.

On-site protocol (warm-up + motor drills) was as follows: 30 reps of rocking motions • 5 laps of bear-walk • 3 laps of crocodile-walk • 3 laps of starfish jumps • 5 sets of side-roll • 5 sets of bridge/handstand (20 s each) • 3 laps of single-leg-hop per leg. Additional animal walks or mat exercises were introduced according to individual skill (detailed protocol in Supplementary Materials S2).

Then, the home program was as follows: starfish or backpack exercise 15 s × 5 sets • ‘Xavier’ backward-fall drill 5 reps × 5 sets. Execution frequency was left to each family’s discretion. (The detailed in Supplementary Materials S2.)

### 2.6. Ethical Considerations

This study complied with the Declaration of Helsinki and was approved by the Academic Research Ethical Review Committee of Waseda University (Approval No. 2023-423). Parents or legal guardians provided written informed consent for participation. Additionally, all participating children provided verbal assent appropriate for their age and understanding. Participation was entirely voluntary, with the right to withdraw at any time. All data were anonymized to ensure confidentiality.

### 2.7. Data Availability

The anonymized datasets (e.g., scores and positive rates of primitive reflexes and evaluations of social behaviors) used in this study are deposited in Dryad and can be accessed via the following DOI:10.5061/dryad.dfn2z35d8. This ensures full transparency and reproducibility of this research.

### 2.8. Statistical Analysis

As a pilot study, no a priori power analysis was conducted. Therefore, the sample size was determined pragmatically based on recruitment feasibility, and findings should be interpreted as exploratory. Raw scores (0–4) were analyzed as ordinal data; additionally, for clinical interpretability, we dichotomized the scale (1–4 = retained, 0 = integrated) when calculating positivity rates. The Friedman test was used to compare residual primitive reflex scores (ranging from 0 to 4). If a significant difference was found, post-hoc analysis was conducted using the sign test to compare pre- with post-four weeks (4W), post-eight weeks (8W), and post-12 weeks (12W). Furthermore, the effect size for non-parametric tests was calculated as *r = Z⁄√N* and interpreted using Cohen’s conventions (small = 0.10, medium = 0.30, and large = 0.50) [28]. For the positivity rate, items with a four-point scale were categorized into positive (1–4) and negative (0), whereas other items were classified as positive (+) and negative (−). The statistical significance of differences in the distribution of positive cases across pre-, post-4W, post-8W, and post-12W was analyzed separately for the ASD and ADHD groups using Cochran’s Q test. If a significant difference was detected, McNemar’s test was conducted for post-hoc analysis. For SRS-2 and Conners 3 total scores, normality was assessed with the Shapiro–Wilk test and Q–Q plot inspection. If the data followed a normal distribution, a paired *t*-test was applied; otherwise, the Wilcoxon signed-rank test was used. Effect sizes were calculated using Cohen’s *d* for the *t*-test and *r* for the Wilcoxon test. Cohen’s *d* was interpreted based on established criteria (small = 0.20, medium = 0.50, and large = 0.80) and calculated as (mean_post—mean_pre)/SD_pooled [28].

No missing data were recorded; hence, all analyses were performed on complete cases. Because this is an exploratory pilot study with a modest sample, no formal adjustment for multiple comparisons was applied; *p* values should therefore be interpreted as descriptive. MCID was estimated by the distribution method (SEM = SD × √(1 − r)); reliability coefficients were set at r = 0.84 for SRS-2 and r = 0.88 for Conners-3, according to the validation studies [26,27]. The significance level was set at *p* < 0.05.

## 3. Results

### 3.1. The 12-Week Change in Primitive Reflex

**Figure 1:**
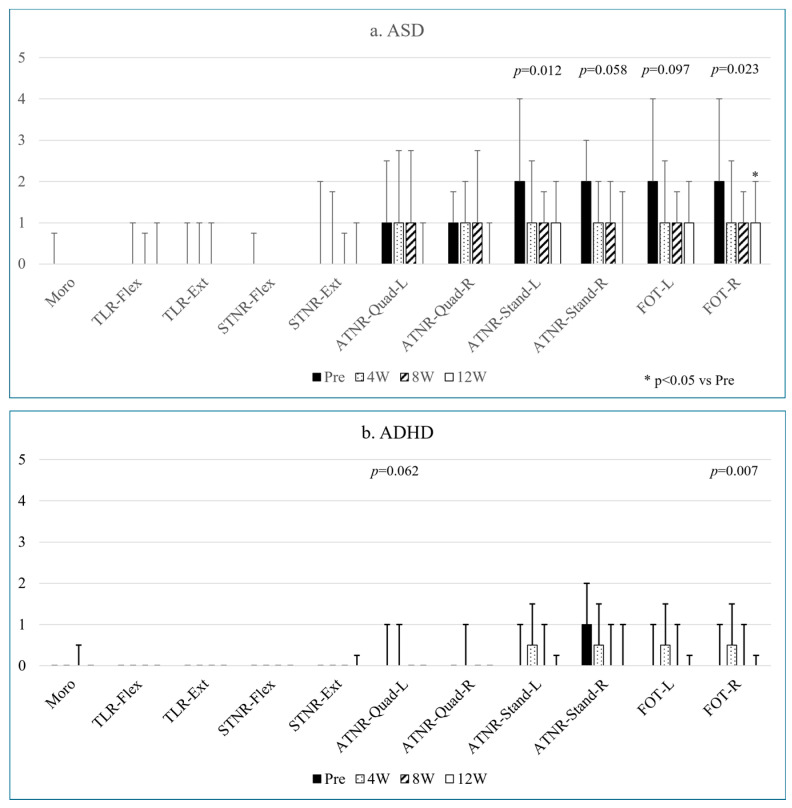
Changes in the persistence levels of various primitive reflexes and FOT in ASD (**a**) and ADHD (**b**) over twelve weeks.

Changes in primitive reflexes and FOT following the 12-week intervention are presented in Figure 1. The effect size and detailed statistical values are available in Table 2a,b and Supplementary Materials S3.

In the case of ASD, ATNR-Stand-L and FOT-R showed decreased retention levels, with a significant pre–post-12W difference in both ATNR-Stand-L (*p* = 0.012) and FOT-R (*p* = 0.023) (Figure 1a). The effect sizes indicated moderate to large differences in ATNR-Stand-L and a large difference in FOT-R (Table 2a). ATNR-Stand-R and FOT-L tended to decrease over twelve weeks, with moderate to large effect sizes for ATNR-Stand-R and small to moderate effect sizes for FOT-L (Table 2a).

In the ADHD group, FOT-R retention levels decreased over twelve weeks (*p* = 0.007), although the post-hoc test did not reveal a significant difference (Figure 1b). ATNR-Quad-L also showed a decreasing trend (*p* = 0.062). The effect sizes were large for FOT-R and small to large for ATNR-Quad-L (Table 2b).

Additionally, large effect sizes were observed for ATNR-Quad-L and ATNR-Quad-R between pre- and post-12-week intervention in the ASD group (Table 2a) and, for FOT-L, between pre- and post-4 weeks and 8 weeks in the ADHD group (Table 2b).

### 3.2. Changes in the Number (And Percentage) of Individuals with Positive Primitive Reflex Retention

Table 3a,b presents the number and percentage of individuals classified by retention levels of various primitive reflexes. Levels 1–4 or ‘+’ were categorized as ‘retention present (+)’, while level 0 or ‘−’ was categorized as ‘no retention (−)’. No significant differences were observed in the distribution of individuals with ASD regarding retention status, except for ATNR-Stand-R (*Q* = 10.24, *p* = 0.017) and Galant (*Q* = 9.32, *p* = 0.025) (Table 3a). Post-hoc analysis revealed that the retention level of ATNR-Stand-R at post-12W was significantly lower than at pre (*p* = 0.031), whereas no significant time-point differences were found for the Galant reflex (Table 3a).

Similarly, in individuals with ADHD, no significant differences were found in retention status distribution except for FOT-R (*Q* = 12.60, *p* = 0.006); however, the post-hoc test did not reveal any significant differences (Table 3b).

Notably, over 50% of individuals with ASD showed retention in the ATNR in both quadruped and standing positions for both sides, as well as the FOT in both hands (Table 2a). Individuals with ADHD exhibited retention in the ATNR-Stand-R and the FOT in both hands (Table 3b).

### 3.3. Three-Month Score Changes in SRS-2 (ASD) and Conners 3 (ADHD)

No statistically significant difference was observed in the SRS-2 total score before and after the 12-week intervention in individuals with ASD (*p* = 0.072); however, a moderate effect size was found (Table 4).

In contrast, the Conners 3 total score and Global Index in individuals with ADHD were significantly lower at post-12 W compared to pre-intervention (*p* = 0.016 and 0.020, respectively) (Table 5). No other significant changes were found in the subscales of SRS-2 or Conners 3.

## 4. Discussion

### 4.1. Summary of Results

This study investigated the effects of a 12-week intervention focusing on rhythm, balance, and whole-body coordination exercises on RPRs and socio-behavioral outcomes in children with ASD and ADHD. The results revealed that ATNR was commonly retained in both groups, but with a broader range and bilateral presentation in children with ASD. Significant reductions were observed in ATNR-Stand-L score in ASD (*p* = 0.012, r = 0.465–0.572), alongside decreased FOT-R score (ASD: *p* = 0.023, r = 0.554–0.623; ADHD: *p* = 0.007, r = 0.546–0.764). The Conners 3 Total Score (*p* = 0.016) and Global Index (*p* = 0.020) showed large, clinically meaningful reductions, as indicated by the MCID (Table 5). Although no statistically significant changes were observed in the SRS-2 total score, a small to moderate effect size was identified, suggesting a possible trend warranting further investigation. These findings suggest that short-term motor interventions may be associated with improvements in selected reflex patterns and behavioral outcomes.

### 4.2. Patterns of Primitive Reflex Retention in ASD and ADHD

Consistent with earlier studies [16,29], our findings revealed broader and more bilateral RPRs in children with ASD compared to those with ADHD. While bilateral ATNR, Galant, and Landau reflexes were common in the ASD group, ADHD participants primarily exhibited right-sided ATNR. Such differences may reflect distinct neurodevelopmental characteristics underlying each condition.

These group differences might be related to differential maturation of neural circuits. Neuroimaging studies suggest that children with ASD may exhibit reduced hemispheric lateralization and atypical long-range connectivity, especially in neural circuits involved in motor coordination and postural control [15,21]. This altered connectivity could potentially contribute to the bilateral persistence of primitive reflexes observed in this group. Although speculative, differences in ATNR laterality may reflect underlying sensorimotor asymmetries in neural function, potentially distinguishing neurodevelopmental profiles in ASD and ADHD. However, this interpretation requires validation through neurophysiological data. This pattern aligns with previous findings suggesting that children with ADHD tend to show higher rates of ATNR and Galant reflex retention compared to typically developing peers [14,30,31]. These studies support the hypothesis that retained primitive reflexes, particularly those associated with vestibular and spinal pathways, may be linked to behavioral dysregulation in ADHD.

It is essential to interpret RPRs cautiously. Prior studies—mostly in preschool children aged 4–6 years and using less stringent cut-offs—report RPRs persistence in approximately 65% of typically developing children [29]. Direct comparison should therefore be made cautiously. Therefore, RPRs should not be treated as pathognomonic of developmental disorders. Instead, their relevance likely depends on the combination of reflex type, laterality, persistence beyond age norms, and association with functional impairments.

### 4.3. Exercise Intervention and Primitive Reflex Integration

The intervention appeared to be associated with statistically significant changes in specific reflexes, particularly in ATNR, as well as in fine motor coordination as measured by FOT. While these findings align with prior studies employing rhythm and cross-pattern movement-based programs [19,32], full integration of RPRs was not consistently achieved, particularly in children with ADHD. One possible explanation may be the relatively low intervention frequency (once weekly), which contrasts with protocols in previous research using thrice-weekly sessions [33].

Exercise programs integrating bilateral motor tasks and sensory input have been reported to facilitate reflex inhibition [20]. Therefore, increasing session frequency and embedding motor–cognitive dual tasks could potentially enhance efficacy in future applications, particularly for children with ADHD who may benefit from more structured and repetitive input to achieve reflex integration.

### 4.4. Socio-Behavioral Outcomes and Mechanisms

The intervention was associated with statistically significant reductions in Conners 3 Total Score and Global Index in the ADHD group, with moderate to large effect sizes. Although no statistically significant changes were found in the SRS-2 scores in the ASD group, the observed effect size suggests potential benefits that require further exploration (Table 4). These findings are consistent with prior meta-analyses indicating small-to-moderate benefits of physical activity on attention and behavior in children with neurodevelopmental conditions [34].

Acutely, exercise has been shown to up-regulate catecholamines and prefrontal activity [35]; evidence for chronic adaptations over ≥ 12 weeks is emerging, with several systematic reviews reporting moderate-to-large effect sizes for physical activity interventions in ADHD [34,36]. Pontifex et al. and Tomporowski et al. suggest that physical activity may acutely modulate executive functions and emotion regulation [35,37]. In ASD, fMRI studies have revealed reduced hemispheric lateralization in networks involved in social communication and motor control, suggesting atypical neural organization [38]. Given this, bilateral motor training has been proposed as a promising avenue to promote interhemispheric integration and functional reorganization, particularly in brain circuits subserving social and sensorimotor functions in ASD.

Given the modest improvements observed, integrating brief, structured movement activities—such as 5-min cross-pattern exercises performed twice per day—into daily routines may provide low-burden support for behavioral self-regulation. For instance, a classroom-based ‘movement break’ might consist of 30 s of bear-walk followed by 30 s of Cross-Crawl, repeated in five cycles (approximately 5 min). Although the current study did not directly test these specific routines, previous studies suggest that short bouts of bilateral movement may acutely enhance attention and executive function in children with neurodevelopmental conditions [35,39].

### 4.5. Limitations and Future Directions

This study has several limitations. First, the small sample size and lack of a control group limit generalizability and preclude causal inference. Second, although all participants completed the 12-week program with high adherence, there was slight variability in weekly frequency and implementation setting (center vs. home). Third, RPRs' evaluations relied on behavioral observation, and although inter-rater reliability was acceptable, objective neurophysiological validation was not conducted.

Future research should adopt randomized controlled designs with higher-frequency interventions and standardized protocols. The use of neuroimaging (e.g., EEG, fMRI, etc.) or sensor-based motion analysis could provide mechanistic insight and improve the precision of outcome measurement. Additionally, examining the role of play-based activities in reflex integration may offer accessible alternatives to structured interventions.

### 4.6. Conclusions

This study provides preliminary evidence that a 12-week rhythm-, balance-, and coordination-focused motor program may facilitate the integration of specific primitive reflexes and contribute to behavioral improvements in children with ASD and ADHD. While the effects were modest and selective, the findings underscore the potential value of incorporating movement-based interventions into early support strategies. Tailoring intervention frequency and content to diagnostic profiles may enhance efficacy, and further research should explore both the neurophysiological and psychosocial pathways—such as executive function, self-regulation, and sensorimotor integration—that may mediate the observed behavioral changes.

## Figures and Tables

**Table 1:** Demographics of participants.

Variable	ASD (*n* = 15)	ADHD (*n* = 12)	Total (*n* = 27)
Age, years, M ± SD	9.4 ± 1.9	9.5 ± 1.7	9.4 ± 1.8
Sex, *n* (M/F)	13/2	8/4	21/6
Comorbidities	None *	None *	None *

* No additional neurodevelopmental or medical comorbidities were reported; exclusion criteria precluded enrollment of children with concurrent diagnoses.

**Table 2:** (**a**): Effect sizes (*r*) for changes in residual levels of various primitive reflexes following a twelve-week exercise intervention: pre–post 4 weeks, pre–post 8 weeks, and pre–post 12 weeks in individuals with ASD. (**b**) Effect sizes (*r*) for changes in residual levels of various primitive reflexes following a twelve-week exercise intervention: pre–post 4 weeks, pre–post 8 weeks, and pre–post 12 weeks in individuals with ADHD.

(**a**)			
ASD	Pre-4 W	Pre-8 W	Pre-12 W
Moro	0.365	0.098	0.336
TLR-Flex	0.071	0.190	0.222
TLR-Ext	0.249	0.000	0.125
STNR-Flex	0.092	0.156	0.082
STNR-Ext	0.067	0.220	0.310
ATNR-Quad-L	0.219	0.209	**0.514**
ATNR-Quad-R	0.389	0.284	**0.516**
ATNR-Stand-L	0.465	**0.572**	0.484
ATNR-Stand-R	0.330	**0.515**	0.496
FOT-L	0.253	0.377	0.432
FOT-R	**0.623**	**0.554**	**0.600**
(**b**)			
ADHD	Pre-4W	Pre-8W	Pre-12W
Moro	0.289	0.500	0.289
TLR-Flex	0.387	0.236	0.387
TLR-Ext	0.289	0.314	0.314
STNR-Flex	0.289	0.387	0.408
STNR-Ext	0.289	0.387	0.000
ATNR-Quad-L	0.129	0.408	**0.545**
ATNR-Quad-R	0.327	0.119	0.327
ATNR-Stand-L	0.109	0.204	0.342
ATNR-Stand-R	0.204	0.333	0.465
FOT-L	**0.546**	**0.557**	0.436
FOT-R	**0.577**	**0.764**	**0.546**

Bold numbers indicate large effect sizes.

**Table 3:** (**a**): Changes in the number and percentage of individuals classified as ‘+’ (or 1–4) in retention levels of various primitive reflexes in individuals with ASD. (**b**) Changes in the number and percentage of individuals classified as ‘+’ (or 1–4) in retention levels of various primitive reflexes in individuals with ADHD.

(**a**)				
ASD	Pre	4W	8W	12W
Moro	4 (26.7)	3 (20.0)	3 (20.0)	2 (13.3)
TLR_Flex	3 (20.0)	5 (33.3)	4 (26.7)	6 (40.0)
TLR_Ext	6 (40.0)	6 (40.0)	5 (33.3)	4 (26.7)
STNR_Flex	5 (33.3)	4 (26.7)	3 (20.0)	2 (13.3)
STNR_Ext	6 (40.0)	6 (40.0)	5 (33.3)	6 (40.0)
ATNR-Quad-L	11 (73.3)	9 (60.0)	10 (66.7)	6 (40.0)
ATNR-Quad-R	11 (73.3)	9 (60.0)	10 (66.7)	7 (46.7)
ATNR-Stand-L	11 (73.3)	10 (66.7)	10 (66.7)	8 (53.3)
ATNR-Stand-R	12 (80.0)	12 (80.0)	10 (66.7)	7 (46.7)
Landau	10 (66.7)	8 (53.3)	10 (66.7)	11 (73.3)
Galant	9 (60.0)	10 (66.7)	7 (46.7)	5 (33.3)
Babinski (L)	6 (40.0)	7 (46.7)	6 (40.0)	6 (40.0)
Babinski (R)	6 (40.0)	6 (40.0)	7 (46.7)	5 (33.3)
FOT-L	11 (73.3)	9 (60.0)	7 (46.7)	5 (33.3)
FOT-R	10 (66.7)	8 (53.3)	7 (46.7)	4 (26.7)
(**b**)				
ADHD	Pre	4W	8W	12W
Moro	1 (7.7)	0 (0.0)	3 (23.1)	0 (0.0)
TLR_Flex	2 (15.4)	3 (23.1)	1 (7.7)	0 (0.0)
TLR_Ext	3 (23.1)	3 (23.1)	1 (7.7)	1 (7.7)
STNR_Flex	0 (0.0)	1 (7.7)	2 (15.4)	2 (15.4)
STNR_Ext	1 (7.7)	2 (15.4)	0 (0.0)	3 (23.1)
ATNR-Quad-L	4 (30.8)	4 (30.8)	2 (15.4)	1 (7.7)
ATNR-Quad-R	2 (15.4)	4 (30.8)	2 (15.4)	1 (7.7)
ATNR-Stand-L	4 (30.8)	6 (46.2)	3 (23.1)	3 (23.1)
ATNR-Stand-R	7 (53.8)	6 (46.2)	4 (30.8)	4 (30.8)
Landau	4 (30.8)	4 (30.8)	3 (23.1)	2 (15.4)
Galant	3 (23.1)	5 (38.5)	6 (46.2)	4 (30.8)
Babinski (L)	3 (23.1)	2 (15.4)	1 (7.7)	3 (23.1)
Babinski (R)	4 (30.8)	3 (23.1)	2 (15.4)	2 (15.4)
FOT-L	7 (53.8)	7 (53.8)	4 (30.8)	6 (46.2)
FOT-R	9 (69.2)	7 (53.8)	3 (23.1)	4 (30.8)

The numbers in parentheses indicate percentages (%).

**Table 4:** Changes in SRS-2 in ASD before and after a 12-week exercise intervention and MCID.

	Pre (S.D.)	Post (S.D.)	ΔPost–Pre (Range)	MCID	*p* Value (Effect Size)
Awareness	9.3 (3.8)	9.4 (3.8)	0.9 (−4.0–4.0)	1.20	0.905 (0.031)
Cognition	15.9 (5.9)	15.5 (5.1)	−0.4 (−7.0–5.0)	1.88	0.596 (0.140)
Communication	25.0 (10.2)	23.3 (7.9)	−1.7 (−13.0–6.0)	3.24	0.158 (0.386)
Motivation	10.7 (3.7)	9.7 (4.1)	−1.0 (−7.0–5.0)	1.17	0.223 (0.329)
RR Behaviors	14.5 (5.4)	13.3 (4.5)	−1.2 (−5.0–6.0)	1.69	0.126 (0.420)
Total Score	77.3 (22.0)	71.3 (18.7)	−6.0 (−27.0–14.0)	6.96	0.072 (0.502)

‘Social-’ is abbreviated in awareness, cognition, communication, and motivation. RR represents ‘restricted and repetitive’. MCID was calculated based on the distribution-based approach using SEM ($SEM=SD\times \sqrt{1-r}$) with *r* = 0.9.

**Table 5:** Changes in Conners 3 in ADHD before and after a 12-week exercise intervention and MCID.

	Pre (S.D.)	Post (S.D.)	ΔPost–Pre (Range)	MCID	*p* Value (Effect Size)
Inattention	16.3 (7.7)	12.8 (7.6)	−3.5 (−25.0–5.0)	3.43	*0.092 (0.486)*
Hyperactivity/Impulsivity	20.8 (10.0)	15.8 (9.8)	−5.0 (−28.0–3.0)	4.47	*0.077 (0.510)*
Learning Problems	10.7 (7.5)	8.8 (7.3)	−1.9 (−11.0–3.0)	3.33	0.180 (0.413)
Executive Functioning	14.1 (6.0)	13.2 (5.7)	−0.9 (−7.0–6.0)	2.69	0.384 (0.262)
Aggression	7.1 (7.4)	5.9 (6.1)	−1.2 (−10.0–5.0)	3.31	*0.623 (0.142)*
Peer Relations	3.1 (2.9)	2.8 (2.6)	−0.3 (−6.0–3.0)	1.28	0.627 (0.144)
Global Index	14.6 (4.9)	10.6 (6.6)	−4.0 (−22.0–2.0)	2.19	*0.020 (0.671)*
Anxiety	15.8 (7.4)	12.8 (5.7)	−2.9 (−14.0–4.0)	3.31	0.094 (0.529)
Anger	16.3 (7.8)	12.8 (7.3)	−3.6 (−22.0–4.0)	3.49	*0.065 (0.532)*
Conduct Disorder	2.8 (3.3)	1.5 (1.5)	−1.3 (−7.0–2.0)	1.49	0.167 (0.427)
Oppositional Defiant Disorder	9.1 (7.5)	7.8 (7.3)	−1.3 (−12.0–5.0)	3.33	*0.301 (0.298)*
Parenting Index	0.5 (0.8)	0.4 (0.7)	−0.1 (−2.0–2.0)	0.36	0.777 (0.084)
Negative Impact	0.6 (1.2)	0.7 (1.2)	−0.1 (−1.0–2.0)	0.52	*0.705 (0.109)*
Total Score	131.6 (57.3)	105.8 (59.2)	−25.8 (−157–14.0)	25.62	*0.016 (0.695)*

MCID was calculated based on the distribution-based approach using SEM ($SEM=SD\times \sqrt{1-r}$) with *r* = 0.8. The value in *Italics* indicates the *p* values of the Wilcoxon test and the effect size of *r*.

## Data Availability

Available at Dryad [10.5061/dryad.dfn2z35d8].

## Supplementary Materials

The following supporting information can be downloaded at: https://www.mdpi.com/article/10.3390/children12080987/s1. S1: Procedures for assessments; S2: Protocols of each exercise; S3: Median and interquartile range (IQR) values corresponding to the data presented in Figure 1a,b.
