# Supplementary material for: Effects of a 12-Week Exercise Intervention on Primitive Reflex Retention and Social Development in Children with ASD and ADHD
Source: Children (Basel). 2025 Jul 28;12(8):987. doi: 10.3390/children12080987 (PMC12384944; doi:10.3390/children12080987)
Supplement: Supplementary file 1 [file children-12-00987-s001.zip › children-3779828-supplementary.pdf]

**Supplementary Materials S1: Procedures for assessments**

I. Ayres Quadruped Test (ATNR Assessment)

Objective: To evaluate whether the asymmetrical tonic neck reflex (ATNR) is retained.

Procedure:

1. Position the subject in a quadruped posture (hands and knees on the floor).
2. Slowly rotate the subject's head to the left (or right) and hold for 5–10 seconds.
3. Return the head to the central position and hold for 5–10 seconds, then rotate to the opposite side.
4. Perform this sequence 4 times for each side.
5. Observe whether the arm and leg on the side opposite to the direction of head rotation flex.
6. The evaluation side (positive or negative) is determined based on the direction the face is turned.

II. Adapted Hoff-Schilder Test (ATNR Assessment)

Objective: To evaluate the influence of the asymmetrical tonic neck reflex (ATNR).

Procedure:

1. The subject stands upright and extends both arms forward.
2. Rotate the subject's head to either the left or right and hold for 5–10 seconds.
3. Return to the original position and perform the same procedure on the opposite side.
4. Repeat this sequence 3 times for each side.
5. Observe whether the arm(s) move in the direction of head rotation or whether the hips move in the opposite direction. Hip movement reflects ATNR in the legs.

III. Symmetrical Tonic Neck Reflex (STNR) Assessment (Flexion and Extension)

Objective: To assess whether the symmetrical tonic neck reflex (STNR) is retained.

Procedure:

1. Position the subject in a quadruped posture (hands and knees on the floor).
2. Slowly flex the neck forward (as if looking between the knees).
3. Observe whether the arms flex or the legs lift (knees extend) (flexion pattern).
4. Next, extend the neck backward (as if looking at the ceiling).

5. Observe whether the lower limbs flex (extension pattern).
6. Repeat the sequence 6 times.

IV. Tonic Labyrinthine Reflex (TLR) Standing Test

Objective: To evaluate whether the tonic labyrinthine reflex (TLR) is retained.

Procedure:

1. Have the subject stand upright with feet together and arms resting straight at their sides.
2. Extend the head backward (as if looking at the ceiling) and close the eyes.
3. Then, flex the head forward (as if looking at the toes), close the eyes, and hold for 10 seconds.
4. Observe whether the body extends excessively.
5. Observe whether the whole body assumes a flexed posture (bending forward).
6. Observe any loss of balance or alteration of balance when the head is flexed or extended.
7. Observe any compensatory changes in muscle tone at the back of the knees or gripping/increased extensor tone in the toes when the head moves through the mid-plane.

V. Moro Reflex

Objective: To evaluate whether the primitive Moro reflex is retained.

Procedure:

1. Position the subject lying supine with knees slightly bent.
2. Flex the arms and lift them upward, keeping the wrists flexed.
3. The examiner supports the subject’s head in a slightly flexed position and asks them to close their eyes.
4. Provide a sensation as if suddenly dropping the head backward slightly.
5. Observe whether the arms reflexively open outward and to what extent. Then, observe whether the arms return in a hugging motion.
6. Observe if the subject is visibly distressed by the procedure. Additionally, note any marked alteration in the subject’s skin color following the procedure (e.g., pallor or reddening).
7. Observe whether the subject is relaxed enough for the head to drop naturally.

VI. Babinski Reflex

Objective: To evaluate abnormalities in the corticospinal tract.

Procedure:

1. Have the subject sit down.
2. Stroke the outer edge of the sole (from the heel to the toes) with a blunt instrument (e.g., a finger or a dull stick).
3. A normal response is for the toes to curl inward (flexion).
4. An abnormal response is the dorsiflexion of the big toe and fanning out of the other toes (Babinski sign).
5. This response is normal in infants but, if observed beyond the age of 2 years, it suggests a central nervous system disorder.

VII. Landau Reflex

Objective:

To evaluate the coordination ability of the trunk and limbs.

Procedure:

1. Have the subject extend both the upper and lower limbs while lying prone and hold the position for 20 seconds (similar to a "Superman" pose).
2. A positive response includes excessive back extension and leg lifting when the head is raised, which suggests immature neurological development or abnormal muscle tone. When the head is lowered, the trunk and legs should flex.

VIII. Galant Reflex

Objective:

To evaluate the persistence of the spinal reflex.

Procedure:

1. Position the subject in a quadruped posture (hands and knees on the floor).
2. Lightly stroke one side of the spine (from the waist to the shoulder) with a finger or pen.
3. A positive response is a lateral flexion (curving) of the body toward the stimulated side.

IX. Finger and Thumb Opposition Test

Objective: To observe fine motor coordination, movement speed, and associated movements, and to identify signs of neurological abnormalities.

Procedure:

1. Have the subject stand upright with feet together.
2. The subject bends one elbow to 90 degrees and holds the hand in front of the face. They should quickly and accurately touch the thumb to each fingertip in sequence and return to the index finger, repeating this movement five times consecutively.
3. Perform the same procedure with the opposite hand.
4. Observe for associated movements in the opposite hand and assess the smoothness of the opposition movement.

**Supplementary Materials S2: Protocols of each exercise**

*Classroom Exercise Program*

1. Rocking Motion (Coordination) - 30 repetitions

1) Assume a quadruped position with the hands and knees on the floor.

2) Move the hips back toward the heels while rounding the back.

3) Shift the body forward until the shoulders align over the hands.

4) Repeat this back-and-forth rocking motion 30 times.

2. Bear Walk (Coordination Exercise) - 5 laps

1) From a quadruped position, lift the knees off the ground, supporting the body with only the hands and feet.

2) Move the hands and feet alternately, walking in a bear-like motion.

3) Complete 5 laps of the designated course.

3. Lizard Walk / Crocodile Walk (Coordination) - 3 laps

1) Lower the body and move forward using the arms, maintaining a position similar to a push-up.

2) Extend one arm forward while pulling the opposite leg forward.

3) Maintain a low-body posture like a crocodile and move alternately.

4) Complete 3 laps of the designated course.

4. Starfish Jumps (Rhythm & Balance) - 3 laps

1) Stand with the arms and legs spread wide.

2) Jump forcefully while extending the arms and legs outward in the air, forming a starfish shape.

3) Land and immediately jump again.

4) Complete 3 laps of the designated course.

5. Pencil Rolling / Side Rolling (Rhythm) - 5 sets

1) Lie on the back with the arms extended overhead, keeping the body straight.

2) Roll sideways using the whole body without twisting.

3) Roll in the opposite direction and repeat alternately.

4) Perform 5 sets.

6. Bridge or Handstand (Coordination & Balance) - 1 set × 20 sec × 5 sets

Bridge

1) Lie on the back, bend the knees, and place the feet on the floor.

2) Place the hands near the shoulders and lift the body using the arms and legs.

3) Hold the position for 20 seconds.

4) Perform 5 sets.

7. Handstand Steps:

1) Use a wall for support and assume a handstand position.

2) Extend the body as much as possible and hold for 20 seconds.

3) Perform 5 sets.

8. One-Legged Hop (Coordination & Balance) - 3 laps per leg

1) Stand on one leg and maintain balance.

2) Hop lightly while moving forward.

3) Complete 3 laps on one leg, then switch to the other leg.

*Home Exercise Program*

Participants were instructed to perform the following exercises at home.

1. Starfish Exercise (Floor) or Backpack Exercise

Starfish Exercise (Coordination)

1) Lie on the back on the floor.

2) Spread the arms and legs wide, forming a starfish-like posture.

3) Hold the position for 5 seconds, then bend the arms and legs toward the navel and hold for another 5 seconds.

4) Repeat for 5 sets.

2. Backpack Exercise (Coordination)

1) Sit in a long-sitting position with a parent, back-to-back, and link arms.

2) Imitate carrying the parent like a backpack by leaning forward.

3) Hold for a few seconds, then return to the original position.

4) Repeat 5 times.

3. “Xavier” Exercise (Falling Backward from Standing) (Balance)

1. Stand upright with the arms crossed in front of the chest.

2. Relax as much as possible and slowly fall backward, allowing a parent to provide support.

3. Repeat 5 times for 5 sets.

**Supplementary Materials S3: Median and interquartile range (IQR) values corresponding to the data presented in Figure 1a,b.**

Table S1. Median [IQR] scores of primitive reflex items in the ASD group (n = 15)

| Reflex | Pre | 4 W | 8 W | 12 W |
|---|---|---|---|---|
| Moro | 0.0 [0.8] | 0.0 [0.0] | 0.0 [0.0] | 0.0 [0.0] |
| TLR-Flex | 0.0 [0.0] | 0.0 [1.0] | 0.0 [0.8] | 0.0 [1.0] |
| TLR-Ext | 0.0 [1.0] | 0.0 [1.0] | 0.0 [1.0] | 0.0 [0.0] |
| STNR-Flex | 0.0 [0.8] | 0.0 [0.0] | 0.0 [0.0] | 0.0 [0.0] |
| STNR-Ext | 0.0 [2.0] | 0.0 [1.8] | 0.0 [0.8] | 0.0 [1.0] |
| ATNR-Quad-L | 1.0 [1.5] | 1.0 [1.8] | 1.0 [1.8] | 0.0 [1.0] |
| ATNR-Quad-R | 1.0 [0.8] | 1.0 [1.0] | 1.0 [1.8] | 0.0 [1.0] |
| ATNR-Stand-L | 2.0 [2.0] | 1.0 [1.5] | 1.0 [0.8] | 1.0 [1.0] |
| ATNR-Stand-R | 2.0 [1.0] | 1.0 [1.0] | 1.0 [1.0] | 0.0 [1.8] |
| FOT-L | 2.0 [2.0] | 1.0 [1.5] | 1.0 [0.8] | 1.0 [1.0] |
| FOT-R | 2.0 [2.0] | 1.0 [1.5] | 1.0 [0.8] | 1.0 [1.0]* |

**p<0.05 vs Pre*

Table S2. Median [IQR] scores of primitive reflex items in the ADHD group (n = 12)

| Reflex | Pre | 4 W | 8 W | 12 W |
|---|---|---|---|---|
| Moro | 0.0 [0.0] | 0.0 [0.0] | 0.0 [0.5] | 0.0 [0.0] |
| TLR-Flex | 0.0 [0.0] | 0.0 [0.0] | 0.0 [0.0] | 0.0 [0.0] |

| | | | | |
|---|---|---|---|---|
| TLR-Ext | 0.0 [0.0] | 0.0 [0.0] | 0.0 [0.0] | 0.0 [0.0] |
| STNR-Flex | 0.0 [0.0] | 0.0 [0.0] | 0.0 [0.0] | 0.0 [0.0] |
| STNR-Ext | 0.0 [0.0] | 0.0 [0.0] | 0.0 [0.0] | 0.0 [0.3] |
| ATNR-Quad-L | 0.0 [1.0] | 0.0 [1.0] | 0.0 [0.0] | 0.0 [0.0] |
| ATNR-Quad-R | 0.0 [0.0] | 0.0 [1.0] | 0.0 [0.0] | 0.0 [0.0] |
| ATNR-Stand-L | 0.0 [1.0] | 0.5 [1.0] | 0.0 [1.0] | 0.0 [0.3] |
| ATNR-Stand-R | 1.0 [1.0] | 0.5 [1.0] | 0.0 [1.0] | 0.0 [1.0] |
| FOT-L | 0.0 [1.0] | 0.5 [1.0] | 0.0 [1.0] | 0.0 [0.3] |
| FOT-R | 0.0 [1.0] | 0.5 [1.0] | 0.0 [1.0] | 0.0 [0.3] |
